# Texas State Medical Association

**Published:** 1886-01

**Authors:** 


### STATE MEDICAL ASSOCIATION.

### PREPARATIONS ON AN EXTENSIVE SCALE FOR A ROYAL RECEPTION TO THE THOUSAND DOCTORS AND AS MANY DRUGGISTS TO MEET IN DALLAS NEXT APRIL.

The Medical Association of Dallas County met recently in preparation for the State Medical Association meeting, to be held in this city next April. The meeting was very large, and its suggestions throughout characterized with an earnest effort looking to a magnificent reception for the great meeting, to which end the following committees were elected:

On Programme—Dr H K Leake, chairman; E M Tilman, J H Smith, Dr J M Pace, C C Gillespie, W W Leake, W L Kellar, Dr George T Veal, Dr A C Graham, W H Prather, J W Gibbs, S R Rogers, J R Meeks, B Gibbs.

On Reception—Dr S D Thruston, chairman; Dr E L Thompson, R V Tompkins, Dr George Veal, J A Ewing, S H Smith, Jeff House, T L Marsalis, E M Kahn R F Eisenlohr, A Davis, W L Crawford, A C Reeves, Judge R E Burke, John H Brown, A H Belo, Thos Field, J D Parsens, Dr W H Sutton, J L Williams, Dr J L Carter, Dr J V Childers, R M Gano, W H Gaston, Dr R H Jones.

On Decoration—Dr A A Johnson, chairman; Dr George T Veal, Dr W H Sutton, Dr. S A McCarty, Miss Katie Cabell, Mrs L Langdeau, Miss Katie Gano, Mrs J R Johnson, Miss Mattie Burford, Mrs. McRosky, Mrs Dr Johnson, Mrs Quillman, Mrs Dr Allen, Mrs Robert Ogden, Mrs Dr Thompson, Mrs E M Kahn, Miss Lizzie Brown, Mrs. Dr Schiff, Miss Miriam Brown, Mrs R F Eisenlohr, Dr Foscue, Mrs Dr Peters, Mrs Judge Henry, Miss Sue Hamilton.

On Finance—W M Fewsom, chairman, Dr S Eagan, Dr Geo T Veal, Alex Sanger, Dr G Schiff, J W Crowdus, W J Kellar,

Dr E L Thompson, W J Betterton, R V Tompkins, Dr J H Morton, Dr Baldwin, J C O'Connor, Dr E H Ayers, W L Griggs, Dr J V Childers, J D Padgitt, S J Howell, B A Hoyt, Dr. J A Ewing.

On Transportation—Dr R W Allen, chairman; Royal Ferris, J A Ewing, J H Smith, L Elliot, Dr H K Leake, Dr S Eagon, Dr L B Schoolfield, J C Food, I Rheinhardt, W H Abrams, E P Turner.

On Halls—Dr E L Thompson, chairman; Dr J H Morton, Dr S A McCarty, John C Story, M H Hickox, Dr H A Mosely, Dr J W Jones, Dr W R Wilson, S B Clowney, Esq, Dr W H Sutton, Dr J H Gibbs, J C Bogel, Dr G E Peters, C B Gillespie, Dr R H Brown, Dr S W Bullitt, L Richenstein, A Schoellkopf.

On Arrangements—Dr R H Clinton, chairman; Dr E L Thompson, Dr S Eagon, Dr H K Leake, Dr S D Thruston and Dr W H Sutton.

In view of the large number of physicians expected in attendance at the State Association's meeting, which is variously estimated between 600 and 1000, the policy of moving early in the matter of preparations due the dignity of the profession is very judicious. The State Pharmaceutical Association meets here on the same date, and it is proposed to unite efforts for the reception of both associations. It is expected that the citizens of Dallas generally, in view of the direct and indirect benefits to flow to this city from the meeting of two such important associations, will put their shoulders to the wheel in order to give the work of preparation a push ahead. While it would take a prophet, or the son of a phophet, to predict the workings of the State Medical Association, it is certain that subjects of national, and probably of international importance, will be up for discussion and action.—*Dallas News.*

Dallas is one of our proudest cities, and the profession stand deservedly high. We can safely assure our readers that a reception will be accorded delegates worthy in every way of them, of the proud city, and of her noble band of physicians. It will doubtless be the largest gathering of the kind ever held in Texas.

Page(s) missing

